# Observations on Animal Heat; in Reply to the Queries of Æmilius, in This Journal for March Last

**Published:** 1819-09

**Authors:** John Down


FOR THE LONDON MEDICAL AND PHYSICAL JOURNAL.

### *Observations on Animal Heat; in Reply to the Queries of* ÆMILIUS, *in this Journal for March last.* By JOHN DOWN, Esq.

IT was discovered by Dr. Crawford, and published in 1781 in his Experiments and Enquiries into the Cause of Animal Heat, that, if an animal was immersed in a medium hotter than the temperature of its body, the blood would return to the right side of the heart, nearly in the same state or colour as it was sent from the left side. Since this time, I believe, no rational theory has been advanced to account for this phenomenon. My enquiries were instituted for the same purpose, although my experiments at this time are incomplete; therefore, in venturing my opinion upon the above queries, I shall not warrant their correctness: but, as this seems a chasm in physiology, and so closely connected with ÆMILIUS's enquiries, that I cannot give a rational reply to them without entering upon the subject of animal heat, I shall state the outlines and deductions drawn from them; and, should they be incorrect, I trust that some of your numerous readers will state their objections, and bring the matter in question fairly before the public. I cannot trespass upon the pages of your Journal to give my experiments in detail, but shall content myself with my general observations upon them. The florid red colour of the blood is acquired in its passage through the lungs, where it changes from venous to arterial. This, I think, is agreed upon by all physiologists; therefore this will be taken for an established fact.

It has also been proved, that the two most important parts of our atmosphere are oxygen and azote, and one part of this is absorbed by the blood in its passage through the lungs, namely, oxygen ; and this absorption converts the venous to arterial blood. This is the florid-red blood spoken of by Æmilius. It might be asked, why should not the blood at the right side of the heart be the same colour as that of the left? (what I mean by this question is this, why should the blood be carbonized, or converted from arterial to that of venous?) However, we know from experience, that the blood undergoes a change in passing from the left to the right side of the heart, equally as great as it does in passing from the right to the left side, through the tissue of the lungs. I must first account for this, and afterwards endeavour to answer the question.

In the first place, I shall enquire what happens in the lungs in the ordinary mode of respiration, in as few words, to be understood, as possible. The blood passes from the right to the left side of the heart through the tissue of the lungs, and is here exposed to a surface of air far more extensive than the whole body. The blood here absorbs a part of the oxygen of respiration from the atmosphere, but the quantity has always appeared to me to vary according to circumstances; and, in all my experiments, before I formed any theory, my attention was arrested by a sensible difference in the consumption of air, from a cold to that of a warm atmosphere: therefore, the quantity consumed depends upon many circumstances, but particularly on that of the temperature of the medium surrounding the body. When my body has been exposed to a temperature below that of the freezing-point, the consumption of air in the lungs has been from sixteen to twenty ounces, by measure, in one minute: on the contrary, when I have entered a room heated by a stove, where the thermometer stood at 170, the consumption of air was comparatively small, and in some instances I could not discover any waste to have taken place in the confined quantity of air breathed: therefore, the inference drawn by me is, that a healthy subject consumes more oxygen in a cold atmosphere than he does in a hot one; and this coincides with the opinions of those who consider animal heat to be kept up by respiration. No theory, that I know of, has been given to the public, more rational than that of Dr. Crawford's, before alluded to, upon the subject of animal heat. I never could procure a reading of this work except by garbled extracts, but it does not appear altogether unobjectionable; for he does not, I believe, positively make out the exact mode in which it is generated. I think this is his theory: "That the blood, in its passage through the lungs, absorbs the latent heat of the atmosphere, and is given off afterwards in circulating through the body." But this theory has of late been attacked as fallacious, owing to some excellent experiments conducted by Mr. Brodie, by keeping up the circulation of blood by means of artificial respiration. The blood, in this case, seemed to be duly oxygenated; yet the heat of the animal sunk faster than that of another, killed at the same time and under the same circumstances, but left to cool of itself. He also proved by these experiments, that the functions of the animal economy once stopped, though the circulation be kept up by artificial respiration, secretion appeared entirely to cease, and the blood returned to the right side of the heart nearly in the same state of *oxygenation* as it was when it escaped from the left. What really appears to take place in the lungs of a living animal, is this: that the blood, as it passes through this organ in the ordinary mode of respiration, absorbs a vast quantity of oxygen, either to oxygenate the blood or to decarbonize it, or probably both. I shall not here dispute this point; but, as a waste of air indisputably does take place, (and we will say, for instance, eight ounces by measure in one minute,) if this eight-ounce measure of oxygen gas be condensed into the blood, or absorbed, *one of two things must of necessity take place ;* that is, the blood must be increased in *temperature* or in *quantity:* it must therefore either be lighter or hotter than it was before it passed through this organ. It is well known that the blood in the lungs is no hotter than it is in other parts of the body; and Mr. Coleman, in his experiments, has stated the blood to be somewhat colder in the left side of the heart than it is in the right. As the blood is no hotter in the lungs or the left side of the heart than it is in other parts of the body, it must, as before observed, be increased in quantity; that is to say, the blood, when oxygenated, takes more space than it did in a venous or carbonous state, and, consequently, the arterial blood is of less specific gravity than that of the venous; and this I believe to be understood by most physiologists.

If these premises be correct, (and they have in part originated from experiments by myself and others,) it will not be a very difficult task to state the manner in which animal heat is generated and given to the system. The functions of the heart and arteries are, to send and convey the blood to all parts of the body, for the growth, nutrition, and secretion of the glands. All the capillary vessels and glands, the liver excepted, that perform the function of secretion, receive their respective fluids through the medium of the arteries. We will bear in mind this to be the florid-red blood spoken of before, which has been rarefied or expanded in the lungs. The function of the glands depends upon two principal things in the animal economy; that is, their being duly supplied with a proper fluid to perform their secretions, and the nervous stimulus to put these glands and capillaries into action. What this nervous stimulus is, physiologists have not explained, although, in the absence of facts, we have abundant conjectures; but such is the state of things, that the glands do not perform their functions unless stimulated by the nerves. In the absence of this stimulus, the blood may therefore circulate through the body without giving off any heat to the system, as was the case in Mr. Brodie's experiments; but he observed the blood returned to the heart nearly the same colour as it left, and at the same time proved that the glands did not perform their functions by secreting their respective fluids.

From these experiments and observations it will appear, that the glands and capillary vessels, in performing their functions of secretion, change the arterial to that of venous blood; and the lungs change that of venous to arterial. We may therefore recapitulate all that has been said in a few words, respecting the subject of animal heat: That the blood, in its passage through the lungs, absorbs a quantity of oxygen gas, and hence it is *rarefied* or *expanded;* when this expansion has taken place, the blood passes on to the arteries, and is conveyed to the glands and capillary vessels; these are brought into action by the nerves, secrete their respective fluids, change and *condense* the blood from arterial to that of venous, and give off the heat to the system. I therefore shall not call this latent heat, but heat by condensation. This axiom of condensation giving out heat is so well understood by chemical philosophers, that I need not enter into any explanation: however, all medical men may not be chemical philosophers; I shall therefore mention one fact familiar to all: Mix equal parts of sulphuric acid and water together, the temperature will be increased to the boiling point. The two fluids mixed together move in and fill a less space than they did in a separate state; therefore, their specific gravity is increased, and heat given off by condensation.

*Leicester; May* 14, 1819.

P.S. ÆMILIUS's questions are not wholly answered; nor could I, without entering upon the subject, why animals, with a double circulation, should be capable of resisting the heat of 212, or more, with little inconvenience, and without suffering their temperature to be raised much above that of the regular heat of their bodies. My paper already has grown too long to enter upon this subject; but I shall, in some future number of your Journal, communicate the result of my experiments and enquiries upon this subject.

